# Biology of adeno-associated viral vectors in the central nervous system

**DOI:** 10.3389/fnmol.2014.00076

**Published:** 2014-09-19

**Authors:** Giridhar Murlidharan, Richard J. Samulski, Aravind Asokan

**Affiliations:** ^1^Curriculum in Genetics and Molecular Biology, School of Medicine, University of North Carolina at Chapel HillChapel Hill, NC, USA; ^2^Gene Therapy Center, School of Medicine, University of North Carolina at Chapel HillChapel Hill, NC, USA; ^3^Department of Pharmacology, School of Medicine, University of North Carolina at Chapel Hill, Chapel HillNC, USA; ^4^Department of Genetics and Department of Biochemistry and Biophysics, School of Medicine, University of North Carolina at Chapel HillChapel Hill, NC, USA

**Keywords:** adeno-associated virus (AAV), viral vectors, gene therapy, neurological disorders, neurodegenerative diseases

## Abstract

Gene therapy is a promising approach for treating a spectrum of neurological and neurodegenerative disorders by delivering corrective genes to the central nervous system (CNS). In particular, adeno-associated viruses (AAVs) have emerged as promising tools for clinical gene transfer in a broad range of genetic disorders with neurological manifestations. In the current review, we have attempted to bridge our understanding of the biology of different AAV strains with their transduction profiles, cellular tropisms, and transport mechanisms within the CNS. Continued efforts to dissect AAV-host interactions within the brain are likely to aid in the development of improved vectors for CNS-directed gene transfer applications in the clinic.

## INTRODUCTION

Numerous congenital disorders exhibit distinct manifestations in the central nervous system (CNS). Loss of functionality in affected cell types within the brain can often be attributed to defects in single genes. For instance, a range of neurological disorders arise from the inability of cells in the CNS to break down metabolic end products [e.g., lysosomal storage disorders (LSDs)]. One such example of LSDs with fatal manifestations includes globoid-cell leukodystrophy (GLD) or Krabbe disease in which mutations in galactosylceramidase leads to accumulation of the toxin “psychosine” in the CNS (Wenger et al., 2002). This disease shows early onset of symptoms like demyelination, astrocyte gliosis etc., and progresses to the death of patients within 2 years of age (Lin et al., 2005). Other examples of LSDs include Fabry disease, Gaucher disease, GM1/GM2 gangliosidosis, mucopolysaccharidoses disorders, Pompe disease, and neuronal ceroid lipofuscinosis amongst others (Boustany, 2013; Simonato et al., 2013).

Another broad category is neurodegenerative disorders, where functionally distinct neuronal subpopulations are lost due to genetic predispositions or environmental toxins. Commonly known examples of this category include dopaminergic (DA) neuronal loss in Parkinson’s disease and GABAergic neuronal loss in Huntington’s disease (Orr and Zoghbi, 2007; Irwin et al., 2013). A common feature for most of these diseases is severe impairment of combinations of cognitive, motor and sensory functions, leading to loss of quality of life and ultimately death of the patients. Gene therapy holds promise for treating these severely debilitating disorders by delivering healthy cargo of genetic information to specific cell types in the CNS. In particular, adeno-associated viruses (AAVs) have emerged as promising tools for clinical gene transfer in a broad range of genetic disorders with neurological manifestations (Gray, 2013). In this review, we have attempted to bridge our understanding of the capsid biology of different AAVs with their properties such as transduction efficiencies, cellular tropism and transport within the CNS.

## RECOMBINANT ADENO-ASSOCIATED VIRAL VECTORS

Adeno-associated viruses are non-enveloped, helper-dependent parvoviruses with an icosahedral capsid architecture ∼25 nm in diameter. AAVs package ∼4.7 kb genome flanked by ∼145 bp inverted terminal repeats (ITRs) on the 5′ and 3′ ends (Bowles et al., 2006). The wildtype AAV (wtAAV) genome is a linear single stranded DNA consisting of two open reading frames (ORFs). AAV ORFs encode four replication proteins (Rep) and three capsid proteins (Cap/VP) and an assembly activating protein (AAP; Agbandje-McKenna and Kleinschmidt, 2011). In addition, wtAAV requires co-infection by adenoviruses or herpes simplex viruses (HSVs) for successful replication and production of viable AAV particles (Bowles et al., 2006). Three advancements have been instrumental in enabling the use of AAV as a recombinant vector for gene transfer applications: (a) the ability to pseudotype AAV vectors by employing AAV capsids of natural or synthetic origin (Gao et al., 2002, 2004; Rabinowitz et al., 2002; Vandenberghe et al., 2009); (b) cloning and characterization of adenoviral helper genes that are minimally required for generation of infectious AAV particles (Xiao et al., 1998); and (c) understanding that ITRs are the only *cis*-acting molecular signature for successful packaging of a transgene within an AAV capsid (Xiao et al., 1997). These streamlined components are now used to manufacture recombinant AAV (rAAV) vectors packaging a broad spectrum of promoter elements and transgene cassettes for different gene transfer applications (Grieger et al., 2006). It is noteworthy that due to the aforementioned discoveries, we are now able to manufacture AAV vectors with minimal contamination of the wildtype virions.

Different AAV serotypes exhibit a range of properties pertaining to antigenicity, *in vivo* tropism and receptor interactions based on their capsid structures (Agbandje-McKenna and Kleinschmidt, 2011). Capsids of different AAV strains bind a spectrum of cell surface glycan receptors and utilize co-receptors for infection (Huang et al., 2014). These differences in capsid-receptor interactions play a major role in determining the regional and cellular transduction efficiencies of AAV strains across different mammalian organs. Continued progress in understanding the biology of AAV infection over the past two decades has provided the scientific and clinical community with an arsenal of AAV strains that offer desirable features for CNS gene transfer (Lentz et al., 2012). In addition to natural isolates, several laboratory-derived AAV strains have been engineered or evolved for specific CNS gene transfer applications. These efforts have yielded novel AAV vectors for targeting (a) glioblastoma cells (Maguire et al., 2010); (b) rat, mouse and human neural stem cells (Jang et al., 2011); and (c) specific regions (piriform cortex and ventral hippocampus) of blood–brain barrier (BBB) compromised rats (Gray et al., 2010). We discuss the existing inventory of AAV vectors and their characterization within the CNS below.

## BIOLOGY OF AAV CELL ENTRY AND IMPLICATIONS FOR CNS GENE TRANSFER

Successful transduction by AAV vectors is contingent on many key steps like cell surface receptor binding, endocytic uptake, endosomal escape, subsequent nuclear entry, capsid uncoating, genome release, second strand synthesis, and subsequent transcription. Surface exposed regions on the AAV capsids dictate the interactions with the host cell surface (Huang et al., 2014). Cell surface glycans have been identified as the preferred primary receptors for many natural AAVs (Asokan et al., 2012). Accordingly, differences in glycan architecture have been attributed to variations in the efficiency of gene transfer by AAV capsids in different organs. AAV serotypes 1, 5, and 6 bind *N*-linked sialic acid (SA), whereas AAV4 is the only natural AAV isolate that binds *O*-linked SA moieties on mammalian cell surfaces (Kaludov et al., 2001; Walters et al., 2001; Wu et al., 2006b). AAV2, three and six bind heparan sulfate (HS) proteoglycans, whereas AAV9 requires N-terminal galactose residues to perform successful gene transfer (Summerford and Samulski, 1998; Handa et al., 2000; Bell et al., 2011; Shen et al., 2011). Direct injection of HS binding AAV2 in the CNS leads to a largely neuronal transduction profile, whereas SA binding vectors like AAV1 and AAV5 perform efficient neuronal and some glial transduction (Bartlett et al., 1998; Davidson et al., 2000; Mandel and Burger, 2004). The preferential neuronal tropism of AAV2 was later identified to correlate with the comparatively larger availability of heparan sulfate proteoglycans (HSPGs) on the surface of neurons than glia (Hsueh et al., 1998; Hsueh and Sheng, 1999). Interestingly, in addition to enabling the neurotropic bias of AAV2, HS binding has also been associated with restriction of the CNS volume that is effectively targeted by AAV vectors.

It is now known that the lysine residue at position 531 on AAV6 capsid plays an indispensable role in HS binding (Kawamoto et al., 2005; Wu et al., 2006a). By creating HS binding and non-binding variants of AAV1 (AAV1E531K) and AAV6 (AAV6K531E) respectively, Arnett et al. (2013) demonstrated antagonistic effect of HS binding on CNS transduction of intracranially injected AAVs. Supporting these point mutation studies, co-injection of safe doses of soluble heparin also led to substantial increase in CNS transduction by AAV2 (Nguyen et al., 2001; Mastakov et al., 2002). On the other hand, N-terminal galactose binding AAV9 is one of the most efficient vectors for CNS gene transfer. AAV9 has been shown to perform extensive neuronal and glial transduction from different routes of injection in small and large animal models (Cearley and Wolfe, 2007; Foust et al., 2009; Bevan et al., 2011; Dayton et al., 2012; Ahmed et al., 2013; Benkhelifa-Ziyyat et al., 2013; Iwata et al., 2013; Yamashita et al., 2013). In addition to important features on the capsid surfaces, efficiency of AAV vector mediated gene transfer can be affected by several post-entry, trafficking and genome-related events within the CNS. Studies pertaining to some of these aspects of AAV biology have been performed within the context of the CNS and discussed later. First we discuss how different AAV strains are influenced by the route of CNS administration below.

## DIRECT AAV ADMINISTRATION INTO THE CNS

Direct injections of AAV into the CNS have been used to achieve high levels of transgene expression across different animal models (McCown et al., 1996; Chamberlin et al., 1998; Tenenbaum et al., 2004; Bockstael et al., 2012). This strategy of AAV vector administration can be broadly classified into intra-cerebrospinal fluid (CSF) administration and intra-parenchymal administration. The CSF plays a multi-functional role by providing nutrients; molecular and physical cues for important processes like stem cell migration; and removal of interstitial solutes from the brain parenchyma (Sawamoto et al., 2006; Iliff et al., 2012). The CSF is housed within the subarachnoid space, cerebral ventricles, cisterna magna and openings under the cerebellum (foramena), and is in close contact with the spinal cord and brain tissue in the rostrocaudal axis (Koh et al., 2005; Lehtinen et al., 2013). Understandably, efficient delivery of reporter/therapeutic transgenes to large areas of the CNS has been achieved using AAV injections into cerebral ventricles, cisterna magna, or intravertebral lumbar puncture (Davidson et al., 2000; Passini and Wolfe, 2001; Fu et al., 2003, 2007; Liu et al., 2005b; Cabrera-Salazar et al., 2010; Glascock et al., 2012; Rafi et al., 2012; Samaranch et al., 2012, 2013; Chakrabarty et al., 2013). Serotypes such as AAV9 and rh.10 exhibit inherently superior ability to spread within the brain parenchyma. These vectors have been used to achieve widespread and long term expression of corrective transgenes from intra-CSF injections toward disease models of spinal muscular atrophy and Krabbe disease (Glascock et al., 2012; Rafi et al., 2012). On the other hand, some AAV vectors exhibit highly cell-specific transduction profiles from intra-CSF injections. For instance, intracerebroventricular (ICV) administration of AAV4 leads to selective targeting of astrocytes in the k zone surrounding the cerebral ventricles (Davidson et al., 2000; Liu et al., 2005a,b). The ependyma consists of adult neural stem cells that have the ability to perform lifelong migration, differentiation and repopulation of functionally defined regions in the brain (Alvarez-Buylla and Lim, 2004). Indeed, targeted delivery of neurogenic cargo, e.g., noggin and brain derived neurotrophic factor (BDNF) packaged in AAV4 has shown long-term rescue of mouse models of severely debilitating CNS disorders like Huntington’s disease (Liu et al., 2005b; Benraiss et al., 2012). In addition to these *in vivo* studies, biophysical analysis of the AAV4 capsid has revealed distinct structural features and low capsid homology among other natural AAV isolates (Padron et al., 2005; Govindasamy et al., 2006).

Due to the advantages offered by the CSF connectivity of the brain and the spinal cord, AAV vector administration has also been extensively characterized through intrathecal injections (ITs). Traditionally, these injections have been performed by exposing the subarachnoid space at the suboccipital cisterna magna region or the intravertebral space at lumbar region. In general, applications requiring enhanced transduction at the motor, sensory and nociceptive neuronal subpopulations [e.g., within dorsal root ganglia (DRG)] utilize lumbar punctures. AAV serotypes 1, 5, 8, and 9 have shown extensive transduction in the spinal cord and DRG neurons from IT injections at the intravertebral lumbar region (Vulchanova et al., 2010; Hirai et al., 2012; Jacques et al., 2012). In an independent study, Snyder et al. (2011) compared IT injections of AAV vectors 1, 6, 8, and 9 for transduction of motor neurons in the spinal cord and brain stem, and reported superior transduction properties of AAV6 and 9. From studies conducted in large animals like pigs and non-human primates (NHPs), a single IT injection of AAV9 has emerged as the candidate procedure for clinical correction of motor neuron disorders affecting the different regions of the spinal cord (Federici et al., 2012; Gray et al., 2013). As with all these studies, it remains to be seen how vectors pseudotyped with these different capsids respond in a human setting and more importantly, in manifestations of human brain disease.

Direct parenchymal injections of AAV vectors in rodents and NHPs have been traditionally used to achieve transduction within, focused, spatio-functionally distinct regions of the brain (Burger et al., 2004; Lin et al., 2005; Cearley and Wolfe, 2006). AAV2 shows minimal ability to spread from the parenchymal site of injection and performs preferential gene transfer in the neurons (Davidson et al., 2000). Unlike other capsid-receptor interactions, the high affinity for HSPGs has been shown to be detrimental to the spread of AAV2 in the brain parenchyma (Mastakov et al., 2002). As discussed earlier, another vector that lacks the ability to spread from the site of intracerebral injection is the NHP isolate AAV4 (Davidson et al., 2000). In another study performed in adult rats, Burger et al. (2004) demonstrated that *N-*linked SA binding AAV1 and AAV5 are superior to AAV2 in terms of spread of transduction from a single parenchymal microinjection into the hippocampus, substantia nigra, globus pallidus, striatum, and spinal cord. Widespread transgene expression was also achieved by parenchymal injections of AAV7, 8 and 9 in rodents (Broekman et al., 2006; Cearley and Wolfe, 2006). On a cellular level, these vectors preferentially transduced neurons in the adult rodents from clinically relevant stereotaxic injections into the hippocampus, thalamus, cortex and striatum (Cearley and Wolfe, 2006). Interestingly, in addition to capsid serotype, other parameters like age of the animal also seem to affect cellular tropism of AAV vectors from direct brain injections. Using ICV injections of AAVs 1, 8, and 9, Chakrabarty et al. (2013) demonstrated, that injections performed on postnatal day 0 (P0) leads to preferential neuronal tropism. On the other hand the same vectors showed neuronal and astrocytic transduction profiles from injections performed on P1 or later. Against this backdrop of AAV isolates and serotypes that have been extensively characterized for their receptor interactions, novel AAV serotypes isolated from human beings – AAVhu32, 37, 11, 48R3; and NHPs – AAVrh.8 and 10 have been evaluated in neonatal and adult rodents (Cearley and Wolfe, 2007; Cearley et al., 2008). Preliminary studies have confirmed the ability of these vectors to perform transduction comparable to AAV9 in rodents, expanding the AAV vector toolkit for CNS gene transfer. For instance, a recent study conducted head to head comparison of AAV 2, 5, 8, and rh.10 for therapeutic delivery of functional “CLN2” transgene in a late infantile neuronal ceroid lipofuscinosis (LINCL) mouse model. Among different serotypes, AAVrh.10 demonstrated comparatively larger spread of transgene expression and restoration of functional levels of the enzyme tripeptidyl-peptidase I, originally lost as a result of mutations in the CLN2 gene. Improvement in motor activities like gait, balance and grip; and amelioration of seizures led to enhanced survival of the treated mice from a single direct brain parenchymal injection (Sondhi et al., 2007). More recent studies evaluating AAVrh.10 administered through different routes in primates have been reviewed in detail in the context of AAV transport within the CNS below.

## INTRAVENOUS ADMINISTRATION OF AAV VECTORS FOR CNS GENE TRANSFER

Systemic administration of vectors has the potential to achieve ubiquitous gene transfer of the CNS from a single injection. Additionally, the minimally invasive nature of intravenous (IV) injections adds value to clinical administration of AAV vectors via the bloodstream. Two major roadblocks currently impede our ability to utilize this technique for therapeutic gene transfer of the CNS. The first major concern is the broad biodistribution of AAV vector particles into off-target tissues such as the liver, spleen and kidneys during IV administration of AAVs. For instance, IV injections of AAV9 achieves exceptional transduction of neurons and glia in rodents and NHPs, but also leads to enrichment of viral genomes (∼10 fold or more) in the liver and spleen as compared to the brain (Gray et al., 2011). Careful optimization and use of safe dosages of AAV vectors can lead to reduced systemic leakage and associated viral clearance due to neutralizing antibodies (Gray et al., 2011). Another approach to reduce peripheral organ toxicity is the occlusion of blood flow into organs like liver and spleen during IV injections of AAVs (Bevan et al., 2011). Clearly, the use of such techniques requires meticulous optimization of complicated surgical procedures during vector administration before being approved for the clinic. However, it should also be noted that several of these techniques are already approved for use with other drugs/treatments in the clinical setting. Another important problem is the inability of the majority of well-characterized AAV vectors to efficiently cross the BBB and transduce cells within the CNS. In order to successfully transduce cells in the CNS, systemically injected virions are thought to undergo receptor mediated transport to cross the brain microvasculature. However, the exact mechanism(s), paracellular or transcellular remain to be determined. Tight junctions in the endothelial cells, astrocytic endfeet and pericytes are known to collectively constitute the BBB (Zhang et al., 2011; Yang et al., 2014). Intra-arterial infusion of mannitol leads to transient opening of the BBB without eliciting any permanent damage (Fu et al., 2003). Short-term disruption of these checkpoints by administration of mannitol led to effective CNS transduction by IV injections of AAV2 which is unable to cross the BBB (Fu et al., 2003; McCarty et al., 2009).

A recent study compared CNS transduction from injections of AAVs 1, 2, 5, 6, 7, 9, Rh.10, Rh.39, and Rh.43 into the superficial temporal vein of neonatal mice (P1). Successful, but differential levels of CNS transduction were reported from all tested vectors (except AAVs 2 and 5; Zhang et al., 2011). Additionally, some leading examples of AAV vectors that have been tested in adult rodents and NHPs include AAVs 8, 9, Rh.8, and Rh.10 (Shen et al., 2013; Yang et al., 2014). These results clearly indicate that many AAV serotypes have been associated with a range of cellular and regional CNS gene transfer properties from systemic injections. In this regard, a better understanding of capsid structural motifs that allow certain AAV strains to traverse the BBB is critical. For instance, using directed evolution, Gray et al. (2010) have engineered two AAV capsids capable of crossing seizure compromised-BBB in rats. The original library of AAVs from which the candidate capsid was isolated included AAVs 1–6, 8, and 9. Careful assessment of the parental and evolved capsid sequences might provide further insights into capsid domains possibly involved in CNS transduction after IV administration (Gray et al., 2010). Along similar lines, peptide motifs have been identified that impart AAV capsids with the ability to cross the brain microvasculature. IV injection of a peptide modified version of the AAV2 packaging β-glucuronidase was used to achieve significant clearance of lysosomal storage burden, leading to cognitive benefits and prolonged survival in a mucopolysacharidoses VII mouse model (Chen et al., 2012). It is noteworthy that IV administration of the corrective transgene packaged in AAV9 capsid was unable to confer therapeutic benefits. It was later identified using fluorescein labeled *Sambucus nigra* lectin that enhanced SA depositions in the MPS VII affected mouse CNS might be detrimental for AAV9-mediated CNS transduction (Chen et al., 2012). Such results demonstrate that the biology of different AAV strains can be affected by specific disease phenotypes that alter the molecular composition(s) of different cell types within the brain.

## AAV TRANSPORT WITHIN THE CNS

Subsequent to vector administration and engagement of cell surface attachment factors such as glycans, AAV vectors appear to undergo interstitial as well as intracellular transport within the CNS. For instance, recent studies in the primate brain have demonstrated that AAVrh.10 displays distinct transduction patterns following different routes of administration (Rosenberg et al., 2014). Of the five routes tested, delivery to parenchyma resulted in more efficient gene transfer than intraventricular or intraarticular routes of administration. Another study in marmosets demonstrated that IV administration of AAVrh.10 is capable of efficient CNS transduction (Yang et al., 2014). These results highlight the potential diversity in AAV vector transport mechanisms not only in the context of brain physiology, but also possibly due to vector serotype, receptor usage and animal models. Although not completely understood, two mechanisms, namely paravascular CSF transport and axonal transport appear to play a role in controlling the spread of AAV vectors within the CNS. It has been established that the paravascular transport of CSF plays a major role in the spread of interstitial fluid (ISF) within the CNS. One of the earliest studies demonstrated that proteins accumulate along highly vascularized regions of forebrain and brainstem within minutes of ICV injections (Rennels et al., 1985). Further, medically induced blood pressure fluctuations have been directly shown to control the spread of nanoparticles including AAVs in the brain (Hadaczek et al., 2006). The brain is distinct from other organs in that it lacks lymphatic circulation (Cserr et al., 1992; Abbott, 2004). To understand compensatory mechanisms, Iliff et al. (2012) performed CNS-injections of differently sized (between 750 da and 2000 kda) molecular tracers. Using compelling visual evidence provided by 2-photon microscopy, the authors concluded that paravascular movement of CSF clears solutes from the CNS (Iliff et al., 2012). Specifically, the para-arterial influx and the paravenous eﬄux of subarachnoid CSF drain accumulations of metabolic end products and other solutes within the brain parenchyma. These results suggest that mechanisms like the CSF transport can possibly play a role in determining the extent of spread of viruses within the CNS. Clearly, understanding the structure-function correlates of AAV capsids and host factors that might dictate their ability to spread in the brain against the backdrop of CNS physiology will be valuable.

Another known pathway that viruses utilize to spread within the CNS is axonal transport post-entry into host neurons. Viruses can travel long distances by getting transported across synaptic connections in various sectors of mammalian central and peripheral nervous system (Beier et al., 2011; Taylor et al., 2012). Over the years, HSV and pseudorabies virus (PRV) have been used to visualize axonal transport and the resulting patterns of viral infections in the CNS milieu (Granstedt et al., 2013). Although accurate neuronal tracing has been achieved using these viruses, a major disadvantage is the loss of gene expression and neuronal death observed in the labeled cells between 5 days and 2 weeks post-infection (Wickersham et al., 2007; Osakada et al., 2011; Rothermel et al., 2013). In case of AAVs, both unidirectional and bidirectional axonal transport has been observed depending on the viral strain (Hollis et al., 2008; Salegio et al., 2013). During retrograde transport, intact virions are taken up at the axonal projections and are transported to the neuronal cell body (soma), where the virus enters the nucleus to perform transduction. Conversely, a successful anterograde transport requires virions to enter the neuronal soma and travel along the length of the axon to finally get released at the projections. The released virions are then free to transduce new cellular subpopulations in the region.

Understandably, directional axonal transport of AAV can be utilized to achieve safe and targeted gene delivery in spatially and functionally distinct neuronal subpopulations. For instance, AAV2 specifically undergoes anterograde transport (Kells et al., 2009; Ciesielska et al., 2011). On the other hand, AAV6 exhibits exclusive retrograde transport in both rat and primate brain (San Sebastian et al., 2013). In addition, AAV9 has been shown to efficiently travel in both anterograde and retrograde directions (Masamizu et al., 2011; Low et al., 2013; Castle et al., 2014b). Specifically, Castle et al. (2014b) visualized dye-conjugated AAV9 vectors during their anterograde and retrograde movements within cultured rat cortical neurons. These studies showed that axonal transport of AAV9 occurs in Rab7 positive late endosomal/lysosomal compartments. Further, cytoplasmic dynein and kinesin-2 were identified as being critical for successful retrograde and anterograde transport, respectively (Castle et al., 2014a,b).

## SAFETY ASPECTS

Recombinant AAV vector genomes display inefficient integration into the host chromosome and predominantly persist in episomal form (McCarty et al., 2004). This reduces the risk of insertional mutagenesis, often associated with other viral vectors like retroviruses (Bokhoven et al., 2009). The vector genomes subsequently require the host cellular machinery to carry out second strand synthesis, transcription and translation (Duan et al., 1998; Nash et al., 2008). Safety aspects pertaining to persistence of AAV vector genomes in the CNS are forthcoming and have been reviewed in general elsewhere (McCarty et al., 2004; Lentz et al., 2012; Dismuke et al., 2013). Another important safety consideration is the observation that rAAV mediated overexpression of non-self transgenes can elicit immune responses due to antigen presentation of the expressed transgene product. For instance, direct primate brain infusion of AAV1 packaging a humanized *Renilla* GFP transgene triggered an immune response against the translated reporter product (Hadaczek et al., 2009). Similarly, a cell mediated immune response and neuronal loss was observed in rats injected with AAV9 vectors packaging the GFP reporter transgene or a human L-amino acid decarboxylase transgene (Ciesielska et al., 2013). More recently, certain AAV serotypes have been shown to undergo systemic leakage resulting in off-target biodistribution in organs like liver and spleen (Gray et al., 2011; Rosenberg et al., 2014; Yang et al., 2014). These preliminary observations in animal models highlight the need to better understand the parameters that determine potential toxicity/biodistribution profiles and immune response in AAV-mediated CNS gene transfer. It is also important to acknowledge that aspects related to manufacturing, downstream processing and purity of AAV vector preparations are critical toward ensuring the safety of AAV vectors. A comprehensive comparison of different viral gene transfer vectors for parameters such as packaging capacity, host chromosomal integration and other biosafety aspects can be found elsewhere (Lentz et al., 2012; Dismuke et al., 2013).

## SUMMARY

As of early 2014, 5.3% of world-wide clinical trials involving gene therapy have utilized AAV vectors (109 ongoing trials; Journal of Gene Medicine). Only a few of these trials are aimed at treating diseases with CNS manifestations. In the current review, we have attempted to provide an overview of various parameters that might play a role in determining the success of AAV mediated therapeutic gene transfer to the CNS. Interactions of AAV vectors with different primary receptors, directional transport and cellular tropisms following different routes of administration are summarized in **Table 1**. Although we were unable to cover every contribution to the field of CNS gene therapy, we hope that the information provided in this review not only highlights potential gaps in our understanding of AAV-host interactions within the CNS, but will assist with continued vector development for CNS-directed gene transfer applications in the clinic.

**Table 1:** Capsid-receptor interactions, transduction profiles, and axonal transport properties of some of the well-characterized adeno-associated viral serotypes in the mammalian CNS.

Serotype	Primary receptor	Intra-CSF or intra-parenchymal administration		Intravascular administration		Axonal transport
		Neuronal transduction	Glial transduction	Neuronal transduction	Glial transduction	
AAV1	α2,3/α2,6 *N*-linked SA	++	+	+	+	A–,R+
AAV2	Heparan sulfate	+	–	–	–	A+,R–
AAV4	α2,3 *O*-linked SA	–	+	–	–	?
AAV5	α2,3 *N*-linked SA	++	+	–	–	?
AAV6	α2,3/α2,6 *N*-linked SA/heparan sulfate	++	–	+	+	A–,R+
AAV8	?	++	++	++	++	A+, R+
AAV9	Galactose	+++	++	+++	+++	A+,R+
AAVRh.8	?	++	++	+++	+++	?
AAVRh.10	?	+++	+	+++	+++	?

*? Receptor usage/axonal transport has not been characterized; + low levels of transduction; ++ moderate levels of transduction; +++ high levels of transduction; – no transduction; ? A+ or R+ (AAV vector undergoes axonal transport in the anterograde (A) or retrograde (R) direction during *in vivo* characterization).*

## Conflict of Interest Statement

The authors declare that the research was conducted in the absence of any commercial or financial relationships that could be construed as a potential conflict of interest.
